# Distributed Pharmacodynamic Architecture in Multi-Component Herbal Formulations: A Flux-Based Framework for Redox-Heterogeneous Diseases

**DOI:** 10.3390/pharmaceutics18030339

**Published:** 2026-03-10

**Authors:** Moon Nyeo Park

**Affiliations:** College of Korean Medicine, Kyung Hee University, Seoul 02447, Republic of Korea; mnpark@khu.ac.kr

**Keywords:** distributed pharmacodynamics, redox flux modulation, multi-component formulation, electrochemical diversity, pharmacodynamic robustness, Ojeoksan (Wu Ji San)

## Abstract

Cancer is increasingly recognized as a systems-level disorder characterized not only by genetic alterations but also by persistent dysregulation of stress-adaptive signaling networks integrating inflammation, metabolism, immune modulation, and cellular plasticity. Within this framework, reactive oxygen species (ROS) function as flux-dependent regulators of signaling fidelity rather than merely cytotoxic byproducts. Therapeutic strategies centered on single high-affinity targets or indiscriminate antioxidant suppression often fail to achieve durable responses in redox-heterogeneous and inflammation-driven malignancies. Multi-component herbal formulations represent chemically diverse systems capable of distributed pharmacodynamic modulation across interconnected signaling nodes and heterogeneous pharmacokinetic exposure profiles arising from multi-constituent absorption kinetics. Ojeoksan (Wu Ji San), a classical East Asian multi-herbal decoction, has accumulated experimental and clinical evidence demonstrating regulatory effects on inflammatory mediators, metabolic homeostasis, mitochondrial stress responses, and immune signaling pathways. Rather than inducing abrupt pathway inhibition, OJS appears to exert graded, parallel modulation across multiple redox-sensitive axes. Here, we reinterpret OJS within a flux-based pharmacological framework, conceptualizing it as a distributed redox-buffering architecture rather than a direct cytotoxic agent. By integrating Korean and Chinese research traditions with systems-level redox modeling and electrochemical perspectives, we propose that multi-component formulations may enhance pharmacodynamic robustness through controlled modulation of ROS amplitude and multi-node buffering while temporally distributing pharmacodynamic signals through multi-component pharmacokinetic synchronization. From a formulation science standpoint, such distributed electrochemical diversity may expand therapeutic tolerance windows and mitigate compensatory pathway escape in chronic inflammatory and therapy-resistant cancers. This perspective supports repositioning multi-herbal formulations as network-aligned pharmacological systems compatible with modern molecular pharmacology formulation-level design principles and rational combination therapy strategies.

## 1. Introduction

### Reframing Multi-Component Formulations Beyond Single-Target Pharmacology

Contemporary pharmacological development has been predominantly guided by a reductionist paradigm, in which therapeutic efficacy is achieved through high-affinity modulation of a single molecular target using chemically defined agents. While this strategy has proven effective for monogenic disorders and pathway-dominant diseases, it frequently encounters limitations in complex, adaptive pathologies characterized by network-level dysregulation, compensatory signaling, and metabolic plasticity, including cancer and chronic inflammatory disorders [1,2,3,4]. In such contexts, therapeutic resistance and limited durability of response often arise not from inadequate target engagement but from intrinsic network redundancy and dynamic rewiring capacity. Increasingly, systems pharmacology has emerged as an alternative conceptual framework, recognizing that drug action is an emergent property of interactions between pharmacological inputs and multiscale biological networks rather than a linear consequence of single-target inhibition [5,6].

Within this systems-oriented perspective, multi-component therapeutic strategies offer a fundamentally distinct pharmacological architecture. Unlike conventional polypharmacy, which frequently suffers from pharmacokinetic mismatch, asynchronous target exposure, and cumulative toxicity, structured multi-component formulations can produce distributed and temporally buffered pharmacological signals through coordinated absorption, metabolism, and systemic exposure dynamics. Traditional multi-herbal decoctions represent a historically evolved example of such distributed pharmacological systems. Although often discussed primarily in ethnopharmacological or redox-biological contexts, their formulation-level characteristics—comprising heterogeneous physicochemical properties, differential absorption kinetics, and multicomponent metabolic processing—suggest a distinct pharmacokinetic–pharmacodynamic (PK–PD) architecture that warrants re-examination within modern Pharmaceutics discourse [7,8,9,10,11].

Ojeoksan (Wu Ji San), a classical multi-herbal prescription extensively studied in Korean and Chinese pharmacological literature, provides an informative case study. Previous investigations have demonstrated its coordinated modulation of inflammatory mediators, metabolic parameters, mitochondrial function, and stress-responsive signaling networks [12,13,14]. However, these effects have predominantly been interpreted through molecular or redox-centered frameworks rather than through formulation-level pharmacokinetic architecture.

In this review, we propose a conceptual reframing of multi-herbal decoctions as distributed pharmacological systems characterized by heterogeneous absorption kinetics, multi-component PK synchronization, exposure buffering dynamics, and pharmacodynamic signal flattening. By integrating insights from systems pharmacology, redox biology, and formulation science, we position OJS not merely as a collection of bioactive phytochemicals, but as a formulation-level architecture capable of stabilizing dysregulated biological networks through controlled, multi-node modulation. This perspective provides a mechanistic foundation for interpreting multi-component herbal prescriptions within contemporary Pharmaceutics, emphasizing formulation-level control, exposure dynamics, and network-oriented therapeutic design rather than isolated target inhibition.

## 2. Formulation-Driven Pharmacokinetic Systems Architecture

Multi-herbal decoctions such as OJS are characterized by inherent heterogeneity in absorption kinetics, arising from the physicochemical diversity of their constituent phytochemicals. Unlike single-entity drugs that display relatively uniform absorption profiles governed by defined solubility, permeability, and metabolic parameters, complex herbal formulations contain large numbers of structurally diverse constituents identified through chromatographic analysis, including flavonoids, alkaloids, phenolic acids, glycosides, and terpenoids, each with distinct physicochemical properties that influence their gastrointestinal absorption and metabolic processing [15,16]. Analytical profiling studies of classical decoctions using high-performance liquid chromatography (HPLC) and ultra-high-performance liquid chromatography–mass spectrometry (UHPLC–MS) have demonstrated the simultaneous presence of dozens to hundreds of individual compounds within a single prescription, reflecting significant diversity in molecular weight, lipophilicity, and polarity across compound classes [15]. These differences translate into variability in permeability, transporter reliance, and first-pass metabolism, such that absorption occurs across multiple kinetic windows rather than as a synchronized peak exposure, with some constituents absorbed early and others later or more slowly due to their intrinsic ADME properties [16]. For example, investigations into compound decoctions structurally comparable to OJS have shown that specific phytochemical subclasses exhibit distinct Tmax and Cmax profiles, reflecting heterogeneous permeability and first-pass metabolism characteristics. In the case of Qingfei Paidu Decoction (QPD), enzyme inhibition studies demonstrated significant modulation of cytochrome P450 (CYP) isoforms, particularly CYP3A, with in vivo pharmacokinetic consequences including prolonged half-life and increased area under the curve (AUC) of co-administered substrate drugs [17]. This finding illustrates that complex decoctions do not merely undergo passive absorption but actively reshape metabolic processing dynamics through interaction with drug-metabolizing enzymes. Such heterogeneity in absorption kinetics has several pharmaceutically relevant implications. Pharmacokinetic studies of multicomponent herbal preparations have shown that, unlike single drugs, complex decoctions contain many different constituents whose systemic exposure profiles can vary widely after oral administration. For example, simultaneous profiling of multiple absorbed components using chromatographic methods such as LC-MS/MS has been considered critical for evaluating pharmacokinetic behavior of Chinese herbal formulae, and such methods routinely reveal that individual compounds within a decoction exhibit distinct absorption and disposition characteristics due to differences in chemical structure, solubility and metabolic susceptibility [18]. Differential susceptibility to metabolic enzymes compounds this heterogeneity; classical decoctions frequently contain constituents that interact with drug-metabolizing systems, and in vitro and in vivo studies have emphasized the need to investigate constituent-specific ADME properties because these properties govern overall exposure and biological impact of multi-component mixtures [19]. Although specific Tmax and Cmax data for individual compounds may vary between formulas, such compositional and metabolic diversity underlies the concept that single decoction preparations do not generate a single, synchronized systemic peak, but rather produce a temporal dispersion of absorption windows where early-absorbing small polar compounds and later-absorbing larger or metabolically transformed constituents contribute at different times to the overall pharmacological signal. From a systems perspective, absorption kinetics heterogeneity should not be regarded as undesirable pharmacokinetic variability but rather as a characteristic feature of complex herbal formulations that enables temporally staggered systemic exposure, potentially broadening the window of biological interaction relative to single drugs with narrow absorption peaks.

### 2.1. Multi-Component PK Synchronization

In contrast to conventional polypharmacy, where multiple single-entity drugs are co-administered without coordination of their absorption and systemic exposure profiles, multi-herbal decoctions provide an intrinsic mechanism for partial pharmacokinetic synchronization among constituents. Conventional combination therapies often suffer from PK mismatches, where differences in solubility, permeability, metabolic clearance, and tissue distribution result in asynchronous plasma concentration–time profiles that limit synergistic action and complicate dosing regimens. Nanotechnology-enabled co-delivery systems have been developed to address such a mismatch by encapsulating multiple drugs within a single carrier to harmonize exposure and reduce differential clearance rates, which can improve therapeutic outcomes relative to free-drug combinations by overlapping exposure windows and modulating release kinetics [20]. Herbal decoctions inherently avoid some of the PK incompatibilities seen in synthetic polypharmacy because their constituents are extracted and administered as a single formulation, creating a shared dissolution phase following oral administration that partially aligns the gastrointestinal availability of multiple components. Moreover, pharmacokinetic synergy among coexisting compounds—a phenomenon where certain constituents enhance the intestinal absorption or reduce the first-pass elimination of others—has been documented in traditional herbal extracts. For example, co-constituent interactions can increase membrane permeability, inhibit drug transporters (e.g., P-glycoprotein), or modulate pre-systemic metabolism, ultimately generating overlapping but staggered plasma profiles among active compounds that may contribute to coordinated systemic exposure and broadened temporal activity [21]. This phenomenon can be interpreted as a form of temporal PK-PD synchronization in multi-component decoctions, in which partially overlapping yet time-shifted exposure patterns collectively shape pharmacodynamic responses across distributed signaling networks.

### 2.2. Exposure Buffering Model

The heterogeneous absorption and elimination of multiple constituents in a herbal decoction can be conceptualized as an exposure buffering model, wherein the composite pharmacokinetic signal is flattened and temporally distributed relative to a single-entity drug. Unlike narrow peak exposures that produce high Cmax and rapid decline—conditions associated with acute pharmacodynamic effects and potential toxicity—the integration of multiple constituents with distinct absorption and elimination rates results in a broadened composite exposure curve. This distributed profile effectively reduces peak amplitude while extending the duration of systemic presence, which can increase the window of biological interaction and buffer the system against abrupt perturbations in signaling networks. Although direct experimental studies on this specific model in herbal decoctions are limited, pharmacokinetic literature supports the general principle that smoothed exposure curves correlate with reduced peak-dependent toxicity and more sustained therapeutic effects. For instance, formulations engineered to provide sustained release or attenuated Cmax demonstrate lower toxicity profiles and more resilient pharmacodynamic outcomes compared with immediate-release counterparts [22,23,24]. Applied to herbal decoctions, the exposure buffering effect emerges as a systems-level consequence of multi-constituent PK dynamics rather than as an engineered release mechanism, and provides a conceptual foundation for understanding how staggered absorption and elimination can modulate network-level responses without generating high-amplitude perturbations.

### 2.3. Pharmacodynamic Flattening Theory

From a pharmacodynamic perspective, single-target drugs often exhibit steep exposure–response relationships (high Hill coefficients), meaning small changes in concentration result in large changes in effect. This sharp transition is associated with high selective pressure on the targeted pathway and can promote adaptive compensatory responses that undermine long-term efficacy. In contrast, multi-node modulation by complex herbal formulations produces distributed, low-amplitude perturbations across multiple targets and pathways. Network pharmacology frameworks describe how individual constituents within a decoction interact with clusters of functionally related proteins, enzymes, and signaling nodes, enabling a flattening of pharmacodynamic responses that reduce the steepness of exposure–effect curves, lower adaptive resistance pressure, and enhance robustness against compensatory rewiring [22,25,26,27]. This flattening theory aligns with systems control principles from engineering and network biology, where distributed control across multiple nodes stabilizes system behavior relative to single-point interventions. While the mathematical characterization of Hill slopes and network dynamics in poly-constituent systems remains an active research area, the conceptual implication is that multi-target engagement leads to shallower, more resilient pharmacodynamic landscapes that better accommodate adaptive biological processes.

### 2.4. Formulation-Level Control

A major challenge in moving multi-herbal decoctions into standardized pharmaceutics is ensuring formulation-level control that meets regulatory expectations for reproducibility, quality, and consistency. Modern analytical technologies such as high-performance liquid chromatography (HPLC), ultra-high-performance liquid chromatography–mass spectrometry (UHPLC-MS), and chromatographic fingerprinting have become essential tools for quality control of multi-component herbal formulations. These approaches facilitate quantitative marker compound analysis, batch-to-batch consistency assessment, and identification of key phytochemical profiles within complex extracts [28,29]. For example, pharmacopoeial standards increasingly require reproducible HPLC fingerprints for herbal products to ensure that marker constituents meet specified criteria across manufacturing lots, which is critical for establishing GMP-compliant phytomedicine production [30]. Moreover, multivariate fingerprinting approaches using metabolomics and chemometrics can quantify dozens to hundreds of constituents simultaneously, enabling robust monitoring of multi-component quality and offering a comprehensive platform to balance standardization with inherent complexity in herbal decoctions. Combined with emerging pharmacokinetics/pharmacodynamics (PK/PD) integration strategies, formulation-level control frameworks provide a foundation for advancing multi-herbal therapies from empirical practice toward evidence-based, quality-driven pharmaceutics.

## 3. Multi-Herbal Decoctions as Distributed Pharmacological Systems

Conventional pharmacological development has largely been guided by a reductionist paradigm in which therapeutic efficacy is achieved through the selective modulation of a single molecular target using a chemically defined agent. While this strategy has proven successful for certain monogenic or pathway-dominant diseases, it shows intrinsic limitations when applied to complex, heterogeneous disorders characterized by network-level dysregulation and adaptive signaling plasticity, such as cancer, metabolic disease, and chronic inflammatory conditions [31].

Systems pharmacology has emerged as a conceptual and methodological framework to address these limitations by viewing drug action as an emergent property of interactions between multiple pharmacological inputs and multiscale biological networks, rather than as a linear consequence of single-target engagement. Within this framework, combination therapies are not merely additive cocktails of individual drugs, but structured interventions designed to reshape system dynamics, modulate signaling robustness, and buffer pathological feedback loops across molecular, cellular, and organismal levels [32].

Multi-herbal decoctions represent a naturally evolved form of such distributed pharmacological systems. Unlike synthetic combination regimens, which often suffer from mismatched pharmacokinetics, asynchronous target exposure, and exacerbated toxicity due to independent drug clearance profiles, herbal decoctions deliver a constellation of bioactive compounds with heterogeneous physicochemical properties through a unified oral formulation. This intrinsic co-administration enables partial coordination of absorption, metabolism, and systemic exposure, producing a flattened and temporally dispersed pharmacological signal rather than sharp, high-amplitude target perturbations [31].

From a pharmacokinetic–pharmacodynamic (PK–PD) perspective, the therapeutic behavior of multi-herbal formulations cannot be adequately described by single-compound exposure–response relationships. Instead, their effects arise from overlapping, low-intensity interactions with multiple molecular targets, metabolic pathways, and regulatory nodes, consistent with principles of polypharmacology and network modulation. Such distributed engagement reduces selective pressure on any single pathway, thereby limiting compensatory rewiring and resistance emergence that frequently undermine single-target therapies [31].

Recent advances in systems pharmacology and pharmacometabolomics provide experimental support for this view. Integrated PK/PD and metabolomic analyses of multicomponent herbal formulas have demonstrated that distinct classes of absorbed constituents exhibit differentiated temporal exposure profiles and correlate with discrete metabolic and signaling adaptations, collectively contributing to system-level therapeutic effects. These findings suggest that the efficacy of herbal decoctions is not driven by a dominant “active ingredient,” but by coordinated modulation of metabolic homeostasis, redox balance, and stress-responsive networks over time [33].

Importantly, the concept of distributed pharmacological systems aligns closely with contemporary formulation science in Pharmaceutics. Nanoparticle-based combination delivery platforms have been developed to overcome the PK mismatch and temporal discoordination inherent to free-drug cocktails by synchronizing multi-drug exposure and release kinetics. Herbal decoctions, while chemically less defined, achieve a functionally analogous outcome through biological complexity rather than engineered carriers, offering a complementary paradigm for achieving systems-level pharmacological control [31].

Taken together, multi-herbal decoctions can be conceptualized as orally administered, network-oriented pharmacological systems that operate through exposure buffering, multi-target engagement, and adaptive signal modulation rather than maximal inhibition of isolated molecular nodes. This systems-level perspective provides a rational foundation for repositioning traditional decoctions within modern Pharmaceutics discourse, not as empirically derived remedies, but as distributed therapeutic architectures optimized for complex disease biology [31,32].

### 3.1. Distributed Redox Modulation by Multi-Component Herbal Systems

Conventional redox-targeted pharmacotherapy has largely been guided by a single-target paradigm, aiming to inhibit or scavenge specific reactive oxygen species (ROS)-associated enzymes or signaling nodes. While this strategy has yielded short-term efficacy in well-defined contexts, it frequently fails in complex, multifactorial diseases such as cancer, chronic inflammation, and metabolic disorders, where redox signaling is distributed across interconnected and compensatory networks rather than confined to isolated molecular targets [34,35].

Single-target redox drugs often disrupt only a narrow segment of oxidative signaling, allowing biological systems to rapidly adapt through pathway redundancy, feedback activation, or metabolic rewiring. In oncological settings, for example, inhibition of a single antioxidant enzyme or redox-sensitive kinase can be bypassed via parallel redox buffering systems, alternative metabolic fluxes, or compensatory transcriptional programs, ultimately contributing to therapeutic resistance and limited durability of response [7,34].

In contrast, multi-component herbal formulas intrinsically operate through polypharmacological mechanisms, simultaneously modulating multiple nodes within redox, inflammatory, metabolic, and immune signaling networks. Rather than exerting maximal inhibition on a single target, these formulations exert distributed, moderate-intensity perturbations across interconnected pathways, thereby reshaping network behavior at the systems level.

Network pharmacology analyses have demonstrated that herbal formulas typically engage clusters of functionally related targets, including redox enzymes, transcription factors, cytokine regulators, and metabolic sensors, forming a coordinated intervention that aligns more closely with the architecture of complex diseases. This distributed targeting strategy reduces selective pressure on individual nodes, lowers the probability of resistance development, and enhances robustness against network compensation [8,9,35].

Importantly, this systems-level modulation distinguishes multi-component herbal formulas from conventional polypharmacy, where multiple single-target drugs are co-administered. While polypharmacy often increases the risk of adverse drug reactions and drug–drug interactions due to uncoordinated pharmacokinetics and off-target effects, herbal formulas exhibit internally structured compatibility shaped by historical empirical optimization and biochemical complementarity [10,11,36].

From a pharmacodynamic perspective, multi-component formulas can achieve therapeutic synergy through convergent regulation of redox-sensitive pathways, including ROS generation, antioxidant buffering, mitochondrial function, and inflammatory signaling, without exceeding toxicity thresholds associated with high-affinity single-target inhibition. This feature is particularly relevant in chronic or adaptive disease states, where sustained modulation rather than acute suppression of redox signaling is required [7,37].

Collectively, these characteristics position multi-component herbal formulas as distributed pharmacological systems, capable of stabilizing dysregulated redox networks through coordinated, multi-layered intervention. This framework provides a mechanistic rationale for their clinical resilience and sets the conceptual foundation for interpreting OJS not as a collection of independent herbs, but as an integrated network-level redox modulator [1,8].

### 3.2. Network-Level Redox Modulation Across Classical Multi-Herbal Decoctions

OJS is a classical multi-herbal decoction traditionally prescribed for conditions characterized by systemic stagnation, inflammation, and stress-related imbalance. From a modern pharmacological perspective, its therapeutic relevance can be reinterpreted through the lens of systems pharmacology, wherein the formula functions as a network-level modulator rather than as a collection of independent bioactive agents [2,3]. Consistent with this systems-level interpretation, several well-characterized Chinese multi-herbal decoctions, including Xiao-Chai-Hu-Tang and Banxia-Xiexin-Tang, have similarly been shown to regulate redox–inflammatory and stress-adaptive networks through distributed, multi-target mechanisms rather than single-pathway inhibition [3,10,38]. Experimental and computational studies have shown that OJS engages a broad spectrum of molecular targets associated with oxidative stress, inflammatory signaling, metabolic regulation, and cellular stress responses. Network pharmacology analyses reveal that its constituent herbs collectively interact with interconnected redox-sensitive pathways, including antioxidant defense systems, inflammatory mediators, and mitochondrial regulatory nodes, supporting a distributed mode of action consistent with multi-target engagement [4,39,40].

Importantly, evidence suggests that OJS does not act as a simple antioxidant that indiscriminately scavenges ROS. Instead, it appears to modulate redox homeostasis, attenuating excessive oxidative stress while preserving signaling-competent ROS required for physiological adaptation. Such balanced regulation aligns with contemporary views that effective redox-targeted interventions should stabilize signaling dynamics rather than abolish oxidative processes altogether [3,41]. This mode of redox buffering parallels observations reported for other classical decoctions, such as Huangqi Jianzhong Tang and Buyang Huanwu Tang, where preservation of signaling-competent ROS and stabilization of redox homeostasis were linked to therapeutic adaptation rather than indiscriminate antioxidant suppression [10,39,40]. Several studies have reported that OJS administration influences endogenous antioxidant systems, including enzymes involved in ROS detoxification and redox buffering, alongside suppression of inflammation-associated oxidative cascades. These coordinated effects suggest a capacity to reshape the redox–inflammation axis at the systems level, rather than through isolated enzymatic inhibition [39,42]. Similar convergence of antioxidant regulation and inflammation-associated oxidative signaling has been documented in network pharmacology analyses of multi-herbal formulations widely studied in China, supporting the concept that formula-level coordination, rather than dominant single constituents, underlies system-level redox control [3,43].

At the network level, pathway enrichment and target clustering analyses indicate that OJS–associated targets converge on stress-adaptive signaling modules, encompassing redox regulation, immune modulation, and metabolic homeostasis. This convergence enables moderate, parallel perturbation of multiple regulatory nodes, thereby reducing reliance on any single pathway and limiting compensatory rewiring that often undermines single-target redox drugs [40,43]. From a pharmacological robustness standpoint, such distributed modulation may confer advantages in complex disease contexts characterized by redox heterogeneity and adaptive plasticity. By avoiding high-affinity inhibition of individual targets, OJS may reduce selective pressure for resistance while maintaining sustained regulation of dysregulated redox networks over time [10,41]. Collectively, these findings support the conceptualization of OJS as a network-level redox modulator, whose therapeutic behavior emerges from coordinated, low-amplitude interactions across redox-sensitive pathways. This framework provides a mechanistic basis for integrating OJS into modern Pharmaceutics discourse, not as a formula defined by single dominant actives, but as a distributed pharmacological system optimized for stabilizing complex stress-adaptive networks [2,8]. In this regard, OJS can be viewed as a representative example within a broader class of multi-herbal decoctions that have been extensively investigated in Chinese pharmacological literature for their capacity to modulate redox, inflammatory, and immunometabolic networks at the systems level [2,3,4,39,40,41,44,45,46,47,48]. In addition, when multiple phytochemicals are co-delivered within a single herbal matrix, coordinated pharmacokinetic exposure patterns may emerge. Such multi-component pharmacokinetic synchronization differs mechanistically from conventional polypharmacy exposure overlap, where independently administered drugs often produce uncoordinated systemic concentration profiles. In contrast, co-delivery of phytochemicals within formulations such as OJS may generate partially synchronized exposure windows that facilitate cooperative pharmacodynamic interactions across interconnected redox-sensitive targets [3,38,42]. To contextualize OJS within a broader class of multi-herbal decoctions that exhibit network-level redox modulation, representative formulas reported in the literature are summarized in Table 1.

### 3.3. Chemical Determinants of Distributed Redox Modulation

#### 3.3.1. Chemical Diversity and Redox Potential Spectrum

Multi-herbal decoctions represent chemically heterogeneous systems composed of structurally diverse phytochemicals, including polyphenols, flavonoids, quinones, terpenoids, and alkaloids. Each class contains distinct redox-active functional groups—such as catechol moieties, conjugated aromatic systems, and electrophilic quinone intermediates—that differ in electron-donating capacity, oxidation potential, and reaction kinetics. Rather than exhibiting uniform antioxidant strength, these compounds collectively span a spectrum of redox potentials, enabling graded modulation of ROS dynamics across multiple biochemical contexts [53]. Electrochemical analyses of catechol-bearing polyphenols demonstrate that subtle structural variations markedly alter oxidation potential and reducing capacity, indicating that chemical diversity translates into a distributed redox potential range rather than a single antioxidant threshold [54]. Such variability allows simultaneous engagement of ROS-generating and ROS-buffering pathways under different oxidative conditions, thereby supporting dynamic redox equilibration instead of indiscriminate radical quenching. Importantly, quinone–catechol interconversion reactions provide an additional kinetic layer of regulation. In the presence of transition metals, redox-active phytochemicals may undergo controlled redox cycling, generating low-amplitude ROS flux that participates in signaling modulation rather than direct cytotoxicity [55]. This controlled cycling highlights that multi-component herbal systems may influence ROS flux amplitude and temporal oscillation, rather than functioning solely as radical scavengers.

Beyond redox cycling, oxidized quinone intermediates can form covalent adducts with nucleophilic residues such as cysteine or lysine, thereby modulating thiol-sensitive redox sensors and downstream signaling cascades [56]. Through such interactions, chemically diverse constituents may influence transcriptional antioxidant programs, inflammatory kinase pathways, and mitochondrial stress responses in a distributed manner. Phenolic subclasses—including flavonoids, stilbenes, lignans, and related conjugated structures—have been shown to regulate apoptosis, cell cycle progression, and stress-responsive signaling networks in addition to classical antioxidant activity [57]. These observations suggest that chemical scaffold diversity inherently broadens the range of redox-sensitive molecular targets engaged by multi-herbal formulations. In chronic diseases characterized by redox heterogeneity and spatially compartmentalized ROS production, single high-affinity antioxidants may oversuppress signaling-competent ROS while failing to stabilize oscillatory redox flux across compartments. In contrast, multi-component decoctions provide a distributed redox buffering architecture that operates across a broader electrochemical and kinetic range, potentially stabilizing redox amplitude without abolishing physiologically required oxidative signaling [53]. Collectively, these considerations support the view that the pharmacological behavior of multi-herbal decoctions emerges not from a dominant antioxidant constituent, but from a chemically encoded spectrum of redox potentials and kinetic properties. Such distributed electrochemical diversity may underlie their capacity to modulate oxidative stress in complex, adaptive disease states where single-molecule interventions often prove insufficient. Future pharmaceutics-oriented studies employing electrochemical techniques such as cyclic voltammetry may further characterize the integrated redox potential spectrum of the complete OJS decoction and clarify how its multi-component composition contributes to distributed redox buffering. In addition, quantitative characterization of ROS amplitude kinetics using time-resolved or compartment-specific ROS measurements will be necessary to determine whether OJS regulates oxidative signaling through amplitude-modulated redox dynamics rather than simple antioxidant suppression. Consistent with this distributed redox buffering concept, OJS and its constituent phytochemicals have been reported to modestly modulate several redox-sensitive signaling hubs, including Nrf2-dependent antioxidant response, NF-κB-associated inflammatory signaling, and MAPK stress pathways, suggesting regulatory tuning of oxidative signaling rather than strong pathway inhibition. Because multi-herbal formulations such as OJS contain chemically diverse redox-active constituents capable of acting on multiple signaling nodes, they may theoretically reduce the emergence of adaptive resistance compared with single-target redox interventions; however, direct comparative experimental validation remains an important direction for future investigation.

#### 3.3.2. Quinone Cycling and ROS Flux Kinetics

Quinone-containing phytochemicals present in multi-herbal decoctions introduce a fundamentally different redox behavior compared with classical chain-breaking antioxidants. Rather than functioning solely as radical scavengers, quinone and phenolic moieties participate in reversible redox cycling, dynamically modulating ROS flux through electron shuttling and semiquinone intermediate formation [58,59]. Extracellular electron transfer studies using anthraquinone analogs such as anthraquinone-2,6-disulfonate (AQDS) demonstrate that quinone redox couples mediate oxygen-dependent oxidation through sequential electron donation, generating superoxide (O_2_•−), hydroxyl radicals (•OH), hydrogen peroxide (H_2_O_2_), and semiquinone intermediates during cycling [60]. Importantly, radical quenching and electron paramagnetic resonance analyses confirmed that semiquinone and superoxide species function as transient reactive intermediates rather than uncontrolled oxidative bursts. This provides mechanistic support for viewing quinone-containing herbal matrices as controlled ROS modulators rather than indiscriminate oxidants.

Within mitochondria, ROS production is tightly coupled to electron transport chain (ETC) kinetics, redox potential, and membrane polarization state. Mathematical modeling of ETC-linked ROS production demonstrates that site-specific ROS generation depends on NADH/NAD^+^ ratio, membrane potential, and electron backpressure at complexes I and II [61]. Notably, reverse electron transfer through succinate dehydrogenase (SDH) exhibits nonlinear “diode-like” behavior, wherein increased driving force does not proportionally increase ROS production but instead reaches threshold-dependent modulation [62]. These findings indicate that quinone redox systems inherently possess flux-limiting properties that buffer excessive ROS propagation.

From a bioenergetic perspective, quinone/quinol systems should not be interpreted as simple mobile two-electron carriers. Structural and thermodynamic analyses suggest that diffusible reactive species (DRS) generated from quinone interactions may participate in localized redox coupling within membrane complexes [63]. This reinforces the concept that quinone cycling operates through distributed electron transfer networks rather than linear antioxidant cascades.

In the context of multi-herbal decoctions, the coexistence of phenolic hydroxyl groups, quinone backbones, flavonoids, and conjugated aromatic systems generates a spectrum of redox potentials. These compounds can donate electrons, accept electrons, or stabilize semiquinone radicals depending on local oxygen tension and metabolic state. Such compositional heterogeneity enables modulation of ROS flux kinetics rather than simple suppression of ROS amplitude. Crucially, ROS-mediated signaling is amplitude- and duration-dependent. Controlled, low-flux ROS pulses sustain adaptive signaling, whereas high-flux accumulation promotes oxidative damage [61]. Quinone cycling within a multi-component phytochemical matrix may therefore function as a kinetic buffer system: transient ROS generation is permitted to sustain signaling competence, while excessive accumulation is limited through parallel antioxidant and radical-stabilizing interactions.

This kinetic buffering framework provides a mechanistic rationale for why multi-herbal formulas may outperform single-target redox drugs in chronic, redox-heterogeneous diseases. Single-molecule antioxidants typically shift global redox tone but cannot dynamically regulate site-specific ROS production thresholds. In contrast, distributed quinone–phenolic networks enable modulation of electron flux, radical lifetime, and ROS diffusion radius.

Accordingly, OJS and other quinone-rich decoctions may be conceptualized not as antioxidant mixtures, but as distributed redox regulators capable of tuning ROS flux kinetics across inflammatory, metabolic, and mitochondrial signaling modules. This flux-centric interpretation aligns multi-herbal pharmacology with contemporary quantitative redox biology and provides a chemically grounded framework for integrating traditional formulas into modern Pharmaceutics discourse.

#### 3.3.3. Multi-Node Redox Buffering vs. Single-Molecule Scavenging

Conventional antioxidant pharmacology has largely focused on single-molecule radical scavengers, assuming that direct neutralization of ROS is sufficient to restore redox balance. However, emerging systems-level analyses indicate that redox homeostasis is governed by distributed enzymatic and metabolic networks rather than by isolated chemical reactions [14,64].

In biochemically structured metabolic systems, flux distribution across interconnected enzymatic steps determines the steady-state concentration and propagation of reactive intermediates. Analytical models of metabolic resource allocation demonstrate that cellular redox states arise from coordinated modulation of multiple enzyme–metabolite pairs, rather than from maximal activity of any single catalytic node [65]. Consequently, perturbation of a single enzymatic reaction can propagate through upstream and downstream metabolites, altering pathway-level flux gradients.

Similarly, constraint-based modeling of energy metabolism in disease contexts shows that compensatory activation of parallel pathways (e.g., glycolysis vs. mitochondrial respiration) determines the net oxidative burden, highlighting that redox phenotypes reflect system-wide redistribution rather than linear inhibition [66]. These findings suggest that redox modulation should be interpreted at the network level rather than through isolated molecular inhibition.

In contrast to single high-affinity antioxidant molecules that indiscriminately scavenge ROS, multi-herbal decoctions introduce chemically diverse constituents capable of interacting with multiple redox-sensitive nodes simultaneously. Systems-level analyses of metabolic flux regulation indicate that redox homeostasis emerges from coordinated modulation across distributed enzymatic networks rather than from maximal suppression of a single reactive intermediate [14,64]. This multi-node interaction may involve moderate modulation of NADPH-dependent systems, mitochondrial electron transport chain dynamics, inflammatory signaling cascades such as NF-κB and MAPK, and endogenous antioxidant enzymes including superoxide dismutase (SOD), catalase (CAT), and glutathione (GSH)-related pathways [54,58]. Mathematical and biochemical modeling studies further demonstrate that mitochondrial ROS production is governed by threshold-dependent electron flux and compartmentalized redox gradients rather than linear oxidant accumulation [13,67]. Redox signaling therefore depends not only on absolute ROS concentration but also on amplitude, duration, and spatial compartmentalization. Computational modeling of electron transport–linked ROS generation confirms that ROS output is nonlinear and dependent on membrane potential and substrate flux, supporting the concept of amplitude-controlled signaling pulses rather than bulk oxidant excess [13,68].

Excessive single-molecule scavenging may therefore disrupt physiologically required ROS signaling necessary for adaptive transcriptional responses. In contrast, distributed multi-node buffering architectures—such as those proposed for chemically heterogeneous decoctions—enable preservation of signaling-competent ROS pulses while limiting transition into high-flux oxidative damage states [14,54]. From a systems pharmacology perspective, chronic redox-heterogeneous diseases are characterized by adaptive metabolic rewiring and compensatory pathway activation. Constraint-based modeling of metabolic network redistribution demonstrates that perturbation of a single node frequently induces compensatory flux redistribution, reducing therapeutic durability [64]. Multi-component decoctions may instead exert moderate modulation across several interconnected nodes, potentially reducing selective pressure on individual pathways and attenuating compensatory escape mechanisms. Within this framework, OJS and related formulations can be conceptualized as distributed redox modulators whose therapeutic behavior emerges from coordinated modulation of interconnected metabolic, inflammatory, and mitochondrial nodes. This interpretation aligns with emerging pharmaceutics perspectives emphasizing network pharmacology, metabolic resilience, and flux-based therapeutic modulation. Thus, multi-herbal decoctions may be interpreted as distributed redox control systems rather than simple antioxidant mixtures. For complex herbal formulations such as OJS, this distributed pharmacodynamic architecture can be standardized and optimized through modern analytical strategies, including chemical fingerprinting (HPLC/LC–MS), network pharmacology-based target mapping, and quantitative quality control of bioactive marker compounds, thereby enabling reproducible composition, dose consistency, and compatibility with modern combination cancer therapies [2,69,70].

#### 3.3.4. Pharmacodynamic Robustness and Formulation-Level Implications

The distributed redox-buffering behavior described above has direct implications for pharmacodynamic robustness at the formulation level. In contrast to single high-affinity inhibitors, whose efficacy often depends on sustained occupancy of a dominant molecular target, multi-component herbal formulations operate through partial, parallel modulation of multiple nodes. This mode of action reduces dependence on a single biochemical bottleneck and may enhance resilience against pathway-specific compensatory rewiring. In redox-adaptive diseases, therapeutic instability frequently arises from nonlinear feedback amplification within mitochondrial and NADPH-dependent networks. Modeling studies of metabolic flux redistribution demonstrate that perturbation of a single node often induces compensatory activation of adjacent pathways, restoring redox equilibrium despite pharmacological inhibition [14,64]. Distributed formulations, by exerting moderate influence across several interconnected nodes, may dampen this compensatory elasticity and stabilize network behavior over time.

From a formulation science perspective, chemical heterogeneity may therefore function not as pharmacological noise but as a stabilizing design feature. Electrochemical diversity among phytochemical constituents allows coverage of a broader redox potential spectrum, generating a graded modulation profile rather than binary inhibition. This spectrum-based modulation aligns with emerging concepts of flux-based pharmacology, where therapeutic success depends on reshaping signal amplitude and distribution rather than eliminating a molecular species. Importantly, translational studies of GMP-standardized phytomedicines such as SH003 demonstrate that multi-herbal formulations can achieve reproducible pharmacological profiles when subjected to compositional authentication and quality control [30]. Clinical evaluations further indicate that such distributed redox–immune modulation can be achieved without dose-limiting oxidative toxicity, supporting the concept of buffered modulation rather than aggressive redox suppression.

These observations suggest that pharmacodynamic robustness in multi-herbal decoctions may derive from three interrelated features: electrochemical diversity enabling graded redox modulation; parallel multi-node engagement reducing compensatory escape; formulation-level compositional stability ensuring reproducible systems effects. Collectively, this framework positions OJS and related decoctions not merely as traditional botanical mixtures, but as distributed pharmacological systems whose therapeutic durability may stem from network-level flux stabilization. Such an interpretation integrates redox systems biology with Pharmaceutics-oriented design principles, including formulation reproducibility, robustness under metabolic heterogeneity, and translational scalability.

### 3.4. Ojeoksan (Wu Ji San) as a Network-Level Redox Modulator

#### 3.4.1. Multi-Component Herbal Formulas vs. Single-Target Redox Drugs

Conventional redox-targeting strategies in cancer therapy have largely focused on single molecular targets, such as antioxidant enzymes or redox-sensitive kinases. While such approaches demonstrate strong target engagement, they frequently induce compensatory signaling responses that restore redox homeostasis and promote therapy resistance. In particular, excessive ROS suppression or forced oxidative stress can paradoxically enhance cancer cell plasticity and CSC survival. In contrast, multi-component herbal formulas represent a fundamentally different pharmacological paradigm. Rather than exerting strong pressure on a single redox node, these formulations induce moderate and distributed modulation across multiple stress-responsive and signaling pathways. OJS was historically formulated to address complex symptom constellations rather than isolated pathological entities, a principle that aligns with modern concepts of network-level regulation in redox biology and stress adaptation [71,72,73,74]. OJS, a classical multi-herbal prescription, exemplifies this systems-level mode of action. Across diverse experimental contexts, OJS has been shown to simultaneously regulate inflammatory mediators, metabolic parameters, and stress-associated biomarkers, without inducing extreme redox imbalance [2,40,75]. It should be noted that current experimental studies primarily report modulation of inflammatory and metabolic signaling pathways associated with redox regulation, while direct experimental analyses of canonical CSC markers or quantitative comparisons with single-target redox drugs remain limited. Therefore, the proposed network-level modulation framework should be interpreted as a systems-level hypothesis derived from available signaling evidence rather than definitive proof of superiority over conventional single-target strategies. From a pharmaceutics perspective, multi-component herbal formulations such as OJS also raise important considerations regarding formulation standardization, bioavailability optimization, and pharmacokinetic variability among coexisting phytochemicals, which will be critical for translating network-level pharmacological effects into reproducible therapeutic formulations. Furthermore, the clinical limitations of conventional antioxidant-based cancer therapies, including inconsistent efficacy and context-dependent redox responses, highlight the complexity of targeting oxidative pathways in oncology. In addition, variability in herbal composition and the possibility of herb–drug interactions represent important translational challenges that require careful standardization, quality control, and pharmacokinetic evaluation in future studies.

#### 3.4.2. Ojeoksan in the Inflammation–Metabolism–CSC Axis

Chronic inflammation and metabolic reprogramming are increasingly recognized as central drivers of CSC maintenance and therapeutic resistance. Redox signaling functions as a critical integrator of these processes, governing cytokine production, mitochondrial activity, and stress-adaptive transcriptional programs. Notably, a consistent feature across OJS-related studies is its capacity to attenuate inflammatory signaling while simultaneously improving metabolic homeostasis. Traditional indications for OJS, including pain syndromes, digestive dysfunction, fatigue, and systemic imbalance, may retrospectively be interpreted as clinical manifestations of dysregulated inflammation–metabolism coupling under chronic stress conditions [3,76,77]. Experimental models of pain, inflammation, fatigue, and metabolic dysregulation have reported reductions in pro-inflammatory cytokines, normalization of energy-related biomarkers, and improvement of tissue resilience following OJS treatment [12,78,79,80,81]. Although most OJS studies do not directly assess CSC markers, the observed suppression of inflammatory stress and metabolic instability can be strongly mechanistically reinterpreted as indirect modulation of the CSC-supportive microenvironment. Given that CSC survival is critically dependent on permissive stress-buffered microenvironments, the inflammation–metabolism–CSC axis provides a unifying framework through which the diverse effects of OJS may be understood. Within this framework, OJS functions not as a CSC-toxic agent per se, but as a regulator of the pathological stress conditions that facilitate CSC persistence and plasticity. This distinction is consistent with ethnopharmacological principles emphasizing restoration of systemic balance rather than eradication of specific cellular subpopulations.

#### 3.4.3. Therapeutic Implications: Overcoming Resistance

Therapeutic resistance remains a major obstacle in cancer treatment, often driven by redox adaptation, metabolic flexibility, and CSC-mediated tumor repopulation [82]. Single-target redox interventions frequently exacerbate these processes by imposing strong selective pressure on cancer cells, thereby accelerating adaptive escape mechanisms [83]. The pharmacological profile of OJS suggests an alternative strategy. By exerting moderate, distributed effects across inflammatory, metabolic, and stress-response pathways, OJS may reduce selective pressure while maintaining long-term stabilization of redox adaptive signaling. This mode of action contrasts with conventional redox drugs that impose binary oxidative or antioxidative stress, and instead aligns with a systems pharmacology approach that prioritizes resilience and signaling normalization. This network-level modulation is conceptually well-suited to counteract resistance mechanisms that rely on pathway redundancy and cellular plasticity [36]. From a translational perspective, OJS may therefore be positioned not as a standalone cytotoxic therapy, but as an adjuvant or preventive intervention aimed at reshaping resistance-prone tumor states. Such an approach aligns with emerging concepts in systems pharmacology and cancer therapy, warranting further investigation into combination strategies and biomarker-guided applications. Studies on OJS collectively delineate a reproducible pharmacological architecture characterized by distributed modulation of inflammatory signaling, vascular microenvironment regulation, stromal–tumor interaction, and metabolic stress adaptation. As summarized in Table 2, experimental investigations span in vivo inflammatory models, co-culture systems, metabolic disease models, and molecular profiling platforms. Although many studies were originally designed in non-oncologic contexts, the underlying biological axes—MAPK/NF-κB signaling, macrophage-derived cytokine regulation, ROS-dependent transcriptional activation, endothelial dysfunction, and IGF-1–mediated stromal communication—directly intersect with cancer-relevant processes including therapy-induced inflammation, angiogenesis, metastatic niche formation, and chemotherapy resistance. Rather than functioning as a direct cytotoxic agent, OJS demonstrates a consistent capacity to modulate microenvironmental stress signaling and immune-associated pathways, supporting its conceptual positioning as a system-level regulator of pathological network stability. Importantly, the reproducibility of its chemical fingerprint across batches further strengthens its translational potential within a formulation-based anticancer development framework.

## 4. Pathological Rationale: Systemic Dysregulation as a Targetable Disease State

### 4.1. Tumor States as Pathological Entities

Cancer progression is increasingly recognized not merely as a consequence of genetic mutations, but as a pathological state characterized by persistent and maladaptive stress signaling. In physiological conditions, ROS function as tightly regulated signaling mediators that coordinate cellular proliferation, metabolism, and immune responses. However, in cancer, sustained oxidative pressure reshapes these signaling networks into a chronic, stress-adapted pathological state rather than a transient adaptive response [69,70]. In this regard, cancer can be conceptualized as a systems-level disorder of redox-regulated signaling thresholds, in which adaptive mechanisms originally evolved for stress tolerance are co-opted to support tumor progression, immune evasion, and therapeutic resistance [86]. Accumulating evidence indicates that both oxidative stress and reductive stress can promote pathological remodeling, depending on context and duration. Excessive ROS may induce DNA damage and genomic instability, whereas prolonged antioxidant dominance may support tumor survival by suppressing redox-dependent cell death programs and enabling metabolic rewiring [87]. Rather than representing simple quantitative excess or deficiency of ROS, malignant redox states reflect miscalibrated signaling amplitudes that shift the balance between proliferation, apoptosis, differentiation, and immune activation [88]. Experimental evidence supporting this interpretation includes observations that tumor cells frequently maintain elevated basal ROS while simultaneously activating antioxidant buffering systems such as glutathione and thioredoxin networks, as well as compartment-specific redox gradients between mitochondria and cytosol. These spatially and temporally heterogenous redox states indicate that cancer progression is governed not by uniform oxidative damage but by dysregulated redox signaling architecture. From a pathological perspective, tumors can therefore be classified as redox-misaligned systems, in which signaling thresholds governing proliferation, apoptosis, and differentiation are persistently shifted. This misalignment has direct therapeutic implications, as it defines tumor states that may be selectively targeted by anticancer agents designed to restore signaling fidelity rather than indiscriminately suppress oxidative processes [53]. This interpretation resonates with ethnopharmacological concepts that define disease as a state of persistent imbalance and maladaptive regulation rather than a fixed molecular defect, thereby emphasizing restoration of systemic signaling integrity as a therapeutic goal [71,72,73,74]. When translated into modern oncological frameworks, this perspective aligns with precision medicine paradigms that stratify tumors not only by genomic alterations but also by functional signaling phenotypes, including redox amplitude, inflammatory tone, and immune responsiveness [30]. This conceptual framework provides a rationale for therapeutic strategies aimed at restoring rather than indiscriminately scavenging ROS [69,70]. Such strategies are particularly relevant in the context of cancer immunotherapy, where redox-dependent modulation of PD-L1 expression, macrophage polarization, and T-cell activation critically influences treatment responsiveness [89]. Recent studies further demonstrate that oxidative stress amplitude directly regulates immune checkpoint signaling and cytotoxic T-cell exhaustion states, reinforcing the importance of redox-calibrated therapeutic strategies in immuno-oncology [90].

### 4.2. Inflammation–Metabolism–Stemness Coupling as a Redox-Driven Pathological Circuit

Chronic inflammation, metabolic reprogramming, and CSC maintenance are no longer viewed as independent pathological features but as interdependent components of a redox-driven disease circuit. Pro-inflammatory cytokines such as TNF-α and IL-6 elevate intracellular ROS levels, which in turn activate kinases and transcription factors, including NF-κB and MAPKs, reinforcing inflammatory signaling loops [91]. In addition to NF-κB and MAPK signaling, oxidative stress-responsive transcriptional regulators such as nuclear factor erythroid 2-related factor 2 (Nrf2) and hypoxia-inducible factor-1α (HIF-1α) function as key redox-sensitive nodes that dynamically respond to intracellular ROS fluctuations, indicating that redox-modulating interventions may regulate the amplitude and duration of ROS signaling rather than simply suppress oxidative pathways [69,70,91]. In vitro and in vivo studies have shown that OJS modulates multiple redox-sensitive signaling hubs rather than functioning solely as a direct antioxidant. For example, OJS has been reported to regulate endothelial inflammatory signaling by suppressing adhesion molecule expression and restoring nitric oxide–dependent vascular homeostasis through activation of the PI3K/Akt/eNOS pathway. In addition, miRNA-mediated regulation of upstream kinases associated with MAPK and NF-κB signaling has also been observed, suggesting that OJS may influence interconnected redox-responsive networks controlling inflammatory and vascular responses rather than acting through simple ROS scavenging mechanisms [75]. This reciprocal amplification establishes a feed-forward inflammatory network that not only promotes tumor growth but also reshapes the immune microenvironment toward immunosuppressive states [92]. Simultaneously, oxidative signaling modulates mitochondrial function and metabolic plasticity, promoting glycolytic shifts or oxidative phosphorylation flexibility that support CSC survival under therapeutic pressure. CSC populations are particularly adept at maintaining strict redox homeostasis via reinforced antioxidant and metabolic buffering systems, allowing them to withstand both chemotherapy-induced oxidative damage and nutrient stress [93]. Experimental observations further indicate that redox-driven inflammatory signaling, including NF-κB activation, can quantitatively influence the coupling between metabolic regulators and stemness-associated transcriptional programs, suggesting that interventions capable of modulating upstream redox-sensitive signaling hubs may simultaneously affect inflammatory tone, metabolic plasticity, and CSC-associated adaptive phenotypes. These redox-adaptive CSC populations are increasingly recognized as key determinants of resistance to immune checkpoint blockade and targeted therapies, underscoring the need for anticancer agents capable of destabilizing such stress-buffered phenotypes [94].

**Figure 1:**
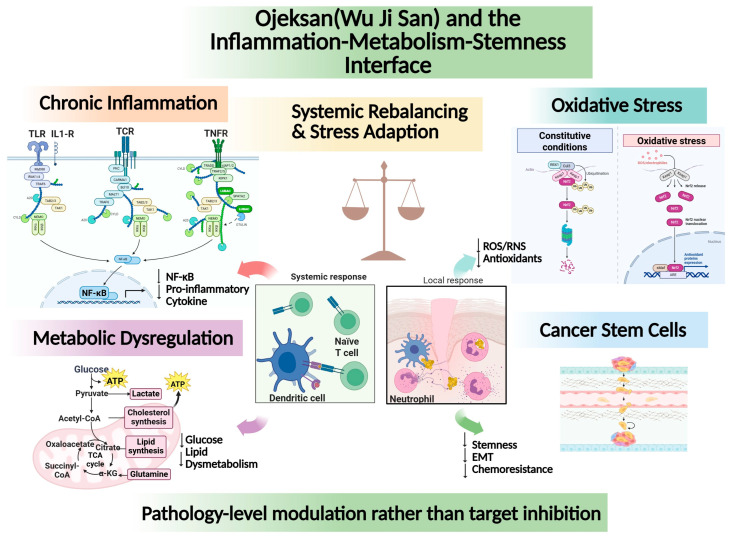
Ojeoksan (Wu Ji San) and the inflammation–metabolism–stemness interface. Schematic representation of the interconnected pathological circuit linking chronic inflammation, oxidative stress, metabolic dysregulation, and CSC-associated adaptive phenotypes. Pro-inflammatory signaling pathways (e.g., NF-κB activation) amplify cytokine production and redox imbalance, while oxidative stress modulates transcriptional and metabolic plasticity. Metabolic reprogramming supports stress-buffered CSC niches that contribute to EMT and chemoresistance. OJS is conceptually positioned as a network-level modulator that attenuates inflammatory signaling, regulates ROS/RNS flux, stabilizes metabolic adaptation, and indirectly constrains CSC-associated phenotypes. Solid arrows represent experimentally supported biological interactions reported in the literature, whereas curved d arrows indicate regulatory feedback or a systems-level integration among pathological modules. The model emphasizes pathology-level modulation rather than single-target inhibition. Abbreviations: Adenosine triphosphate (ATP); Cancer stem cell (CSC); Concentration maximum (Cmax); Epithelial–mesenchymal transition (EMT); Nuclear factor kappa-light-chain-enhancer of activated B cells (NF-κB); Reactive oxygen species (ROS); Reactive nitrogen species (RNS); Toll-like receptor (TLR); Tumor necrosis factor receptor (TNFR).

Evidence from OJS studies demonstrates that this herbal formula suppresses NF-κB activation, reduces inflammatory cytokine signaling, and attenuates downstream proliferative and migratory responses in vascular and inflammatory models [93]. These findings support the interpretation that OJS acts upstream of inflammation–metabolism–stemness coupling by modulating stress-sensitive signaling nodes, rather than targeting a single effector molecule. Pathologically, this mode of action suggests that OJS intervenes at the level of disease-permissive signaling environments, interrupting the reciprocal reinforcement among inflammation, metabolic adaptation, and CSC-associated resistance phenotypes [3,76,77]. Within an anticancer drug development framework, such upstream modulation may function as an immune-normalizing and microenvironment-stabilizing strategy, potentially enhancing the efficacy of cytotoxic agents and cancer immunotherapies [95]. From a therapeutic perspective, this network-level modulation differs fundamentally from conventional single-target anticancer strategies, which often trigger rapid adaptive resistance through pathway compensation. By simultaneously influencing multiple redox-sensitive signaling nodes associated with inflammation, metabolic plasticity, and stemness maintenance, multi-component formulations such as OJS may reduce the likelihood of signaling escape and thereby improve the durability of therapeutic response in the complex tumor microenvironment. This systems-level interference with inflammatory–metabolic coupling may be particularly relevant for inflammation-high or therapy-induced stress phenotypes, which represent clinically actionable subgroups in precision oncology [88]. The integrated interplay among chronic inflammation, oxidative stress, metabolic dysregulation, and CSC-associated adaptive phenotypes is schematically summarized in Figure 1, illustrating the proposed network-level interface through which OJS may exert pathology-level stabilization rather than single-pathway inhibition.

### 4.3. Network-Level Modulation as a Pathology-Guided Therapeutic Strategy

Conventional anticancer therapies are largely designed around single-target inhibition, assuming linear causality between a molecular lesion and disease outcome. However, the pathological state of cancer is intrinsically non-linear and network-based, rendering such reductionist approaches insufficient for durable disease control [96,97]. This limitation has prompted increasing interest in multi-target anticancer agents capable of modulating interconnected signaling circuits rather than isolated molecular nodes [98]. Multi-component herbal formulations, such as OJS, offer a fundamentally different pathological intervention paradigm [99]. By simultaneously influencing multiple stress-responsive signaling nodes—including kinases, transcription factors, and metabolic regulators—these formulations align more closely with the systemic nature of cancer pathology. Network pharmacology analyses further support the notion that multitarget modulation can dampen compensatory signaling routes that frequently underlie therapy resistance [96].

Importantly, this systems-oriented modulation may be particularly advantageous in precision medicine settings, where tumors are stratified according to functional network vulnerabilities rather than single-gene mutations. Importantly, this pathology-guided approach does not imply indiscriminate polypharmacology. Instead, it reflects structured modulation of convergent disease circuits, particularly those governed by redox signaling fidelity. From an ethnopharmacological standpoint, such structured modulation reflects long-standing principles of prescription design aimed at harmonizing interacting pathological processes rather than suppressing isolated disease manifestations [85,100,101,102]. Emerging evidence highlights that natural products possess immunomodulatory activities that influence key elements of antitumor immunity, including checkpoint modulation, macrophage polarization, and T-cell/NK cell activation. These effects can augment current immunotherapy outcomes by mitigating tumor-driven immunosuppression in the tumor microenvironment [103]. In this context, multi-component redox modulators may act as immune-context–dependent anticancer agents that recalibrate microenvironmental signaling thresholds rather than directly inducing tumor cytotoxicity [53]. When appropriately contextualized, multi-component redox modulators may therefore complement conventional therapies by reshaping the tumor microenvironment and reducing the emergence of resistant cellular states [93,96]. Taken together, Section 4 reframes OJS not as an empirical traditional remedy but as a rational, pathology-aligned intervention targeting stress-adaptive systemic dysregulation in cancer. In cancer pharmaceutics development, integrating natural product platforms into precision immuno-oncology frameworks requires functional stratification of tumors and targeted modulation of immune-metabolic pathways. This translational approach leverages multi-omics and network pharmacology to prioritize agents with complementary mechanisms, thus improving therapy synergy and reducing resistance [104].

## 5. Translational Development Strategy for Multi-Component Redox Modulators

**Figure 2:**
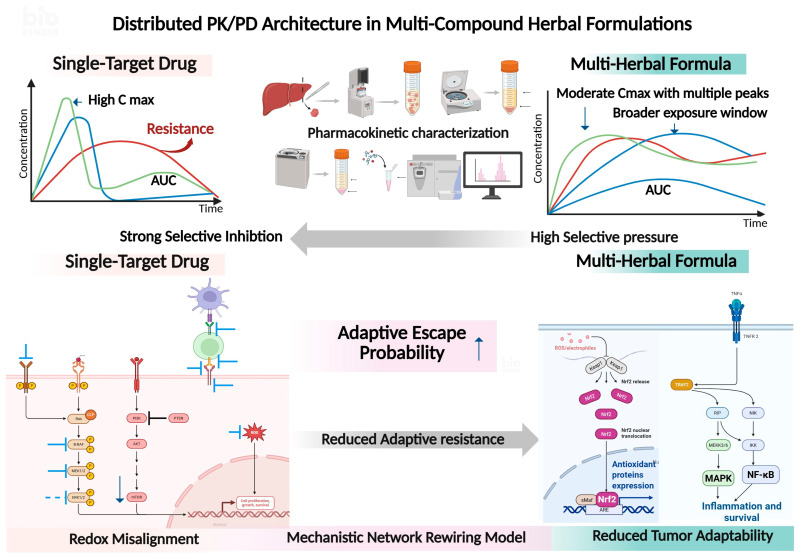
Distributed PK/PD architecture of multi-component redox modulators in cancer pharmaceutics. Schematic comparison between conventional single-target drugs and multi-component herbal formulations. Single-target drugs typically generate high peak concentrations (Cmax) and strong pathway inhibition, which may impose intense selective pressure on cancer cells and promote adaptive resistance. In contrast, multi-herbal decoctions are conceptually expected to generate moderate Cmax with temporally distributed exposure profiles and broader pharmacokinetic windows, enabling parallel modulation of multiple redox-sensitive signaling networks. This distributed pharmacodynamic regulation affects interconnected pathways involved in inflammation, immune signaling, metabolic homeostasis, and redox balance. As a result, multi-component modulation may reduce selective pressure and limit adaptive resistance by distributing regulatory effects across multiple network nodes. The schematic also illustrates the translational pipeline of multi-herbal formulations, from experimental characterization and pharmacokinetic analysis to preclinical validation, clinical evaluation, and regulatory review within a pharmaceutics development framework. Abbreviations: Area under the curve (AUC); Maximum plasma concentration (Cmax); Mitogen-activated protein kinase (MAPK); Nuclear factor erythroid 2–related factor 2 (Nrf2); Nuclear factor kappa B (NF-κB); Pharmacodynamic (PD); Pharmacokinetic (PK).

Effective translation of multi-component redox modulators from mechanistic hypotheses to clinically actionable anticancer agents requires an integrated framework that spans chemical standardization, PK/PD alignment, biomarker-guided stratification, advanced preclinical models such as organoids, and rational combination therapy designs. This section proposes such a structured development strategy tailored to the complex biology of cancer and the multi-target nature of natural product-based formulations. This distributed PK/PD architecture contrasts with high Cmax-driven single-axis inhibition and instead supports network-level modulation within translational development pathways (Figure 2).

### 5.1. Chemical Standardization and Quality Control

Robust drug development begins with chemical standardization of the candidate formulation to ensure reproducibility, safety, and regulatory compliance. Natural products and multi-component herbal extracts show substantial chemical diversity, which necessitates detailed characterization through chromatographic fingerprinting, quantitation of bioactive markers, and good manufacturing practice (GMP)-aligned quality systems. Standardization facilitates consistent PK/PD behavior and comparative analyses across studies, minimizing batch variability that can confound translational efforts.

### 5.2. PK/PD Integration for Redox Modulators

Translational success is predicated on coherent integration of pharmacokinetic (PK) exposure profiles with pharmacodynamic (PD) responses relevant to cancer biology. For redox-modulating agents, PD readouts may include modulation of oxidative stress biomarkers (e.g., ROS levels, redox enzyme activity), inflammatory signaling, and downstream cell fate decisions such as apoptosis or senescence. Coupled PK/PD modeling enables prediction of effective exposure windows, identification of target engagement metrics, and informs dosing regimens that balance anticancer activity with systemic safety. Incorporating PK/PD strategies early accelerates decision-making from preclinical to first-in-human studies. Mechanism-based translational PK/PD modeling, integrating exposure–response relationships with redox biomarker dynamics and immune activation signatures, may enhance prediction of proof-of-mechanism in early-phase trials and reduce Phase II attrition risk [105].

### 5.3. Immunomodulatory Mechanisms of Natural Products in Cancer

Natural products have emerged as rich sources of anticancer agents not only through direct cytotoxic effects but also via immunomodulation—including checkpoint regulation, macrophage polarization, and effector T/NK cell activation. Compounds such as curcumin, resveratrol, and polysaccharide fractions from medicinal fungi demonstrate potent modulation of key immune pathways and tumor microenvironment components in preclinical models [103]. The PD-1/PD-L1 axis, a dominant immune checkpoint exploited by tumors to evade cytotoxic T-lymphocyte activity, is influenced by natural products that can downregulate PD-L1 expression or disrupt its membrane localization, thereby potentially enhancing immune recognition. In parallel, modulation of macrophage polarization toward a pro-inflammatory M1 phenotype and stimulation of NK cell cytotoxicity have been documented in traditional 2D models [103,106]. Given these diverse mechanisms, natural products can function as immune adjuvants or modulators that reshape tumor immunobiology, offering a mechanistic rationale for their integration into combination strategies with immunotherapies. This immunomodulatory spectrum justifies their inclusion in translational development pipelines that seek strategic synergy with immune checkpoint inhibitors and other immune-targeted therapies.

### 5.4. Advanced Preclinical Platforms: Organoids and Organoid–Immune Co-Cultures

Conventional 2D cell culture and animal xenograft models have limited predictive value for human clinical outcomes due to a lack of architectural complexity, stromal microenvironment, and species-specific immune dynamics. By contrast, patient-derived tumor organoids (PDOs) retain the histological and genetic heterogeneity of primary tumors and more faithfully recapitulate tumor physiology [107]. Organoids have therefore risen as powerful platforms for preclinical drug screening, precision oncology, and biomarker discovery. Furthermore, recent advances have enabled organoid–immune co-culture systems, where PDOs are maintained alongside immune populations such as T cells, NK cells, and macrophages, thereby enabling evaluation of tumor–immune interactions in a physiologically relevant context [103,108]. As discussed in recent comprehensive analyses, although conventional organoid studies with natural products focus on direct cytotoxicity, systematic investigation of natural product-mediated immunomodulation in organoid-immune co-cultures remains largely unexplored [109]. This highlights a critical translational gap and a strategic opportunity for integrating immunomodulatory natural compounds into models that better predict human response. Organoid platforms thus anchor translational evaluation by linking in vitro mechanistic endpoints (e.g., cytokine profiles, checkpoint molecule expression, immune cell cytotoxicity) with clinically relevant outcomes. Integration of immune checkpoint blockade within organoid–immune co-culture platforms enables functional assessment of combination strategies and provides mechanistic validation of immune-sensitizing natural products in a patient-specific context [110].

### 5.5. Biomarker Stratification and Precision Oncology Integration

Precision oncology aims to match therapeutic interventions with tumor-specific vulnerabilities. For natural product-based therapies, biomarker stratification can enhance translational success by identifying patient subsets most likely to benefit. Several categories of stratification are pivotal: Inflammation-High Tumors: Tumors with elevated baseline inflammatory signatures often exhibit immune suppression and may benefit most from adjunct immunomodulatory agents that reset microenvironmental immune balance. Metabolically Reprogrammed Tumors: Tumor metabolic phenotypes, especially those characterized by dysregulated redox homeostasis, present mechanistic targets for redox-modulating compounds. CSC-enriched Phenotypes: Cancer stem-like cell populations contribute to therapy resistance and relapse; natural products with evidence of targeting CSC markers offer a rationale for stratification based on stemness signatures. Integration of such biomarker categories into early translational stages allows development of precision-guided clinical hypotheses, adaptive designs, and mechanistic stratification in trial cohorts.

### 5.6. Combination Therapy Synergies

Given the multifaceted biology of cancer and compensatory resistance mechanisms, monotherapy approaches often fail to achieve durable responses. Translational pipelines should therefore incorporate rational combination designs that leverage natural product-mediated mechanisms. With Immune Checkpoint Blockade: Natural products that modulate PD-1/PD-L1, CTLA-4 pathway dynamics, or macrophage polarization may enhance responsiveness to approved checkpoint inhibitors, expanding the subset of responsive patients. With Targeted Therapies: Synergies may also exist between redox or metabolic modifiers and targeted agents (e.g., kinase inhibitors), particularly in tumors with co-regulated signaling networks. Preclinical evaluation of such combinations within organoid–immune co-cultures and stratified organoid panels enables mechanistic synergy assessment and informs candidate prioritization for clinical testing. Early-phase combination trials should incorporate biomarker-informed adaptive designs and real-time molecular profiling to optimize dose selection and minimize overlapping toxicities [111].

### 5.7. Translational and Regulatory Pathways

For natural product-based anticancer candidates to progress toward clinical application, structured translational pathways must integrate rigorous quality control, standardized models, and regulatory engagement. Chemical Standardization: Multi-component extracts require detailed chemical profiling and reproducibility standards, including fingerprinting and quantitation of active constituents. Model Validation: PDO and organoid–immune co-culture models validated against patient response data serve as translational bridges that strengthen clinical hypotheses. Regulatory Alignment: Recent developments, such as the FDA Modernization Act 2.0, recognize advances in vitro models for regulatory decision-making, enhancing acceptance of organoid-based evidence. Efforts to define clinical endpoints that leverage preclinical biomarkers (e.g., cytokine modulation, immune infiltration indices) will facilitate more efficient and informative early trials.

## 6. Conclusions and Future Perspectives

Cancer is increasingly recognized as a systems-level pathological condition characterized by persistent dysregulation of redox signaling, inflammatory circuits, metabolic plasticity, and immune surveillance. Therapeutic strategies limited to single molecular targets often fail to provide durable responses in such dynamically adaptive disease states. In this review, OJS has been repositioned as a pathology-guided, multi-component redox modulator that acts at the level of stress-adaptive signaling environments rather than through direct tumor cytotoxicity. By recalibrating inflammatory, metabolic, and immune-associated signaling thresholds, OJS exemplifies a distributed pharmacodynamic architecture that may reduce the likelihood of compensatory resistance mechanisms.

Importantly, this systems-oriented mode of action situates OJS within contemporary anticancer drug development paradigms that emphasize immunomodulation, microenvironmental regulation, and precision-guided therapeutic integration. Its potential relevance lies in functional tumor stratification contexts—such as inflammation-high, metabolically reprogrammed, or stemness-enriched phenotypes—where network-level stabilization may complement immune checkpoint blockade and targeted therapies. Future translational efforts should therefore integrate rigorous chemical standardization, mechanism-based PK/PD modeling, biomarker-guided patient selection, and advanced preclinical platforms, including organoid–immune systems. Through structured pharmaceutics-driven development strategies, multi-component redox modulators may emerge as precision-oriented adjuncts within modern integrative oncology.

## Figures and Tables

**Table 1:** Representative Multi-Herbal Decoctions as Network-Level Redox and Inflammatory Modulators.

Decoction (Formula)	Representative Experimental/Disease Context	Core Network-Level Actions	Redox- and Immune-Related Mechanisms	Refs.
Xiao-Chai-Hu-Tang (Sho-saiko-to)	Colorectal cancer models, gastric ulcer, stress-associated disorders	IL-6–JAK2–STAT3 signaling, metabolic reprogramming	Suppression of ROS-linked inflammation, reduced glycolysis, restoration of T-cell activity	[4,10]
Banxia Xiexin Decoction	Colitis, intestinal mucosal inflammation	Gut–immune axis regulation	ROS–inflammation buffering, enhancement of IgA and defensin responses	[3]
Huangqi Jianzhong Tang	Gastric ulcer, NSAID-induced mucosal injury	NF-κB/JAK–STAT signaling modulation	Upregulation of antioxidant enzymes (SOD, CAT, GSH) and reduction in lipid peroxidation (MDA)	[49]
Xuefu Zhuyu Tang	Neuroinflammation, traumatic brain injury, tumor-associated microcirculatory dysfunction	Hypoxia–ROS–microcirculation coupling; miRNA–neurotrophic signaling	Redox-linked suppression of TNF-α, IL-1β, IL-6; modulation of oxidative–inflammatory cascades; regulation of miRNA–BDNF–TrkB signaling axis promoting synaptic plasticity and stress adaptation	[50,51]
Liu Junzi Tang	Cancer-associated fatigue, gastrointestinal dysfunction	Metabolic–immune coupling	Attenuation of oxidative stress and maintenance of immune homeostasis	[52]
Shenling Baizhu San	Intestinal barrier dysfunction and immune impairment	Microbiota–redox axis modulation	ROS buffering and stabilization of gut microbiota composition	[43]
Ojeoksan (Wu Ji San)	Chronic inflammatory conditions and cancer-adjunct contexts	Multi-pathway network integration	Coordinated modulation of ROS–NF-κB–MAPK signaling axes	[2]

Abbreviations: Brain-derived neurotrophic factor (BDNF); Catalase (CAT); Glutathione (GSH); Immunoglobulin A (IgA); Interleukin-1 beta (IL-1β); Interleukin-6 (IL-6); Janus kinase–signal transducer and activator of transcription (JAK–STAT); Malondialdehyde (MDA); Mitogen-activated protein kinase (MAPK); MicroRNA (miRNA); Nuclear factor kappa B (NF-κB); Reactive oxygen species (ROS); Superoxide dismutase (SOD); Tumor necrosis factor alpha (TNF-α); Tropomyosin receptor kinase B (TrkB).

**Table 2:** Experimental Evidence Supporting Distributed Redox and Microenvironmental Modulation by Ojeoksan (Wu Ji San).

Pathological Axis/Biological Process	Experimental Model	Key Molecular Findings	Pathological Implication Relevant to Cancer	Refs.
Inflammation-driven stress signaling	Cisplatin-induced acute kidney injury (mouse)	Suppression of MAPK (ERK, JNK, p38) and NF-κB signaling; reduced TNF-α, IL-1β, IL-6	Attenuation of inflammation-associated tissue stress that may contribute to therapy-induced damage and resistance	[2]
Vascular inflammation and endothelial dysfunction	ApoE^−^/^−^ atherosclerosis model	Reduced endothelial inflammation; restoration of eNOS signaling; modulation of Akt pathway	Regulation of vascular microenvironment linked to tumor angiogenesis and metastatic niche formation	[75]
Cell migration and extracellular matrix remodeling	TNF-α–stimulated vascular smooth muscle cells	Inhibition of ROS-dependent NF-κB activation; downregulation of MMP-2 and MMP-9	Suppression of migration-associated pathways relevant to invasion and metastasis-like processes	[40]
Adipocyte-mediated tumor microenvironment and drug resistance	Ovarian cancer–adipocyte co-culture; platinum-treated xenograft	Reduced adipocyte-derived IGF-1 signaling; restoration of platinum sensitivity	Direct modulation of stromal–tumor interaction contributing to chemotherapy resistance	[78]
Inflammation-associated tumor-supportive niche	Colitis-associated colorectal cancer model	Decreased macrophage-derived TNF-α and ERK signaling; alleviation of inflammation-related pain	Regulation of inflammatory tumor microenvironment rather than direct cytotoxicity	[79]
Macrophage-mediated inflammatory signaling	Experimental colitis and nociception models	Attenuation of macrophage-driven inflammatory responses independent of direct tumor killing	Supportive evidence for microenvironmental and immune-modulatory effects	[12]
Formulation reproducibility and chemical consistency	UHPLC–MS/MS profiling of Ojeok-san	Identification and quantitative consistency of 22 marker compounds across batches	Ensures translational reliability of mechanistic and biological findings	[80]
Gastrointestinal disorder	Rat GI motility model	↑ gastric transit, ↓ spasm	Regulation of visceral smooth muscle signaling, autonomic balance	[71,72]
Cold-stress/Qi-stagnation model	Stress-induced rat model	Symptom relief, ↓ stress markers	Stress-adaptive signaling normalization, neuro-endocrine modulation	[3]
Inflammation	Acute inflammation mouse model	↓ edema, ↓ inflammatory mediators	Suppression of inflammatory cascades (upstream stress signaling)	[74,76]
Metabolic disorder	Diet-induced metabolic model	Improved metabolic indices	Regulation of metabolic homeostasis, stress-response pathways	[77]
Inflammation	LPS-activated macrophages	↓ cytokines, ↓ inflammatory mediators	Inhibition of inflammatory transcriptional activation	[3]
Digestive & pain syndromes	TCM clinical cohorts	Symptom improvement	Holistic regulation of multi-organ functional imbalance	[84,85]

Abbreviations: ↓ downregulation, ↑ upregulation, ATP, Adenosine triphosphate; CSC, Cancer stem cell; CFA, Complete Freund’s adjuvant; COX-2, Cyclooxygenase-2; GI, Gastrointestinal; IL-1β, Interleukin-1 beta; iNOS, Inducible nitric oxide synthase; LPS, Lipopolysaccharide; NF-κB, Nuclear factor kappa B; NO, Nitric oxide; RAW264.7, Murine macrophage cell line; ROS, Reactive oxygen species; TCM, Traditional Chinese medicine; TNF-α, Tumor necrosis factor alpha; UHPLC, Ultra High Performance Liquid Chromatography.

## Data Availability

No new data were created or analyzed in this study.

## Abbreviations

AMPK	AMP-activated protein kinase
AKT	Protein kinase B
CSC	Cancer stem cell
ETC	Electron transport chain
HIF-1α	Hypoxia-inducible factor 1 alpha
IL-1β	Interleukin-1 beta
IL-6	Interleukin-6
KEAP1	Kelch-like ECH-associated protein 1
MAPK	Mitogen-activated protein kinase
MS	Mass spectrometry
mtROS	Mitochondrial reactive oxygen species
NADPH	Nicotinamide adenine dinucleotide phosphate
NF-κB	Nuclear factor kappa B
NRF2	Nuclear factor erythroid 2–related factor 2
OXPHOS	Oxidative phosphorylation
PPP	Pentose phosphate pathway
PI3K	Phosphoinositide 3-kinase
ROS	Reactive oxygen species
TCA cycle	Tricarboxylic acid cycle
TCM	Traditional Chinese medicine
TNF-α	Tumor necrosis factor alpha
UHPLC	Ultra-high-performance liquid chromatography
